# Supplementary material for: Local-scale determinants of arboreal spider beta diversity in a temperate forest: roles of tree architecture, spatial distance, and dispersal capacity
Source: PeerJ. 2018 Sep 18;6:e5596. doi: 10.7717/peerj.5596 (PMC6149511; doi:10.7717/peerj.5596)
Supplement: Supplemental Information 1 [file peerj-06-5596-s001.docx]

**Supplemental information**

**Table S1** The significance of tree architecture, spatial distance, dispersal capacity and joint effects on spider beta diversity.

| **Fractions of variation** | ***R*^2^_adj_** | ***F*** | ***P*-value** |
| --- | --- | --- | --- |
| [a+b+f+g] Environmental + joint | 0.135 | 12.21 | 0.001 |
| [b+d+e+g] Spatial + joint | 0.159 | 5.21 | 0.001 |
| [c+e+f+g] Dispersal capacity + joint | 0.097 | 16.44 | 0.001 |
| [a+b+c+d+e+f+g] | 0.284 | 5.53 | 0.001 |
| [a] Environmental alone | 0.052 | 4.51 | 0.001 |
| [b] Spatial alone | 0.077 | 2.78 | 0.001 |
| [c] Dispersal capacity alone | 0.068 | 13.86 | 0.001 |
| [d+g] Spatially structured environmental | 0.087 | - | - |
| [d] Residual | 0.716 | - | - |

**Table** **S2** Decomposition of the variation in beta diversity patterns attributing to spatial, environmental and joint effects for two spider groups with contrasting vagility.

|  | **With common fraction** | |  | **Without common fraction** | |
| --- | --- | --- | --- | --- | --- |
|  | **Spatial effects** | **Environmental effects** |  | **Spatial effects** | **Environmental effects** |
| High | 0.180*** | 0.165*** |  | 0.073*** | 0.058*** |
| Low | 0.135*** | 0.111*** |  | 0.078*** | 0.053*** |

Notes: *P*-values were calculated through 1000 random permutations, *** <0.001.
