# Supplementary figures and images for: Local-scale determinants of arboreal spider beta diversity in a temperate forest: roles of tree architecture, spatial distance, and dispersal capacity
Source: PeerJ. 2018 Sep 18;6:e5596. doi: 10.7717/peerj.5596 (PMC6149511; doi:10.7717/peerj.5596)

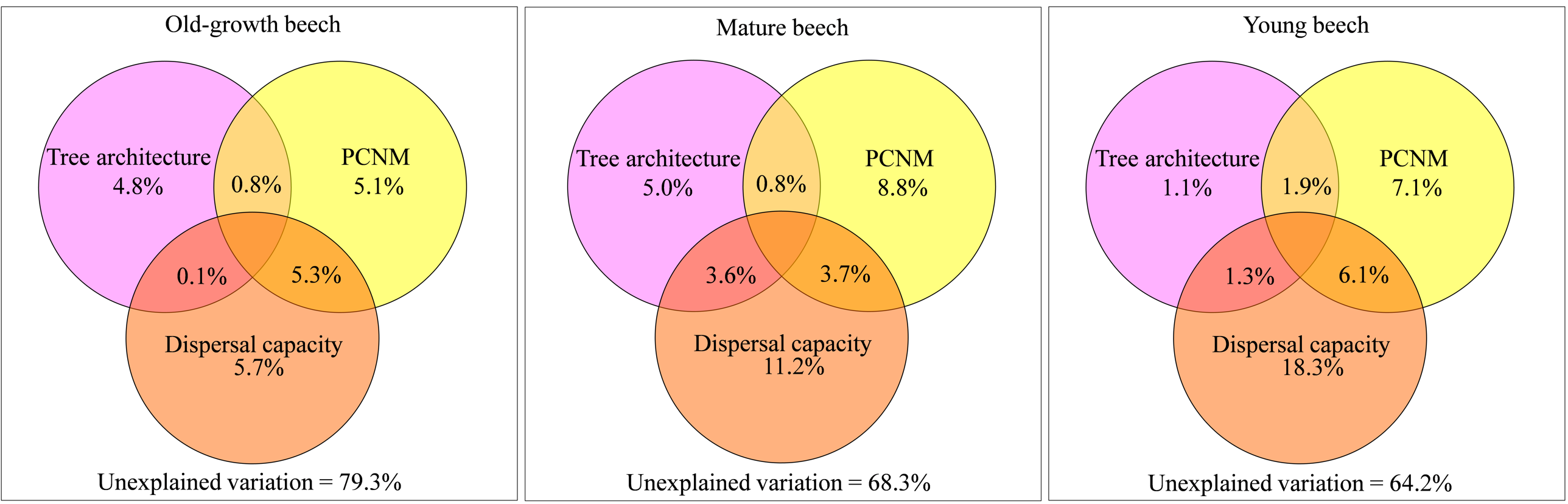

Supplement: Figure S1 [file peerj-06-5596-s002.png]
